# The Effects of High-Frequency Transcutaneous Electrical Nerve Stimulation for Dental Professionals with Work-Related Musculoskeletal Disorders: A Single-Blind Randomized Placebo-Controlled Trial

**DOI:** 10.1155/2015/327486

**Published:** 2015-11-17

**Authors:** Hye Rim Suh, Tae Hoon Kim, Gyeong-Soon Han

**Affiliations:** ^1^Department of Physiology, College of Medicine and Neuroscience Research Institute, Korea University, Seoul 02841, Republic of Korea; ^2^The Post-Professional DPT Program, Stockton University, Galloway, NJ 08205-9441, USA; ^3^Department of Dental Hygiene, Gachon University College of Health Science, Incheon 21936, Republic of Korea

## Abstract

Work-related musculoskeletal symptom disorders (WMSDs) have a significant issue for dental professionals. This study investigated the effects of high-frequency transcutaneous electrical nerve stimulation (TENS) on work-related pain, fatigue, and the active range of motion in dental professionals. Among recruited 47 dental professionals with WMSDs, 24 subjects received high-frequency TENS (the TENS group), while 23 subjects received placebo stimulation (the placebo group). TENS was applied to the muscle trigger points of the levator scapulae and upper trapezius, while placebo-TENS was administered without electrical stimulation during 60 min. Pain and fatigue at rest and during movement were assessed using the visual analog scale (VAS), pain pressure threshold (PPT), and active range of motion (AROM) of horizontal head rotation at six time points: prelabor, postlabor, post-TENS, and at 1 h, 3 h, and 1 day after TENS application. Both groups showed significantly increased pain and fatigue and decreased PPT and AROM after completing a work task. The TENS group showed significantly greater improvements in VAS score, fatigue, PPT, and AROM at post-TENS and at 1 h and 3 h after application (all *P* < 0.05) as compared to the placebo group. A single session high-frequency TENS may immediately reduce symptoms related to WMSDs in dental professionals.

## 1. Introduction

Work-related musculoskeletal symptom disorders (WMSDs) develop as a result of repetitive movements, awkward posture, and/or external force. WMSDs decrease the professional's skills, work output, job performance, and quality of life [1, 2]. Various studies have described interventions and workplace ergonomic designs for workers with WMSDs [3, 4]. However, no controlled environmental factor by the diversity of the subjects' occupations might affect the results of these studies. Therefore, it is important to study a single type of occupational group for the confirmation of accurate treatment effects.

The prevalence of the WMSDs in dental professionals, such as a dentist or a dental hygienist, is comparatively high because of their work environments that require sustaining a certain position or the overuse of certain muscles. The resulting physical load places them at risk for the occurrence of musculoskeletal disorders [5, 6]. WMSDs in dental professionals are accompanied by decreased quality of life and restricted participation in social activities as well as decreased quality of patient care [7, 8]. Appropriate interventions for dental professionals with WMSDs are thus essential to improve the quality of life [9]. However, despite the importance of effective therapeutic interventions, the appropriate management of WMSDs in dental professionals remains unclear.

Transcutaneous electrical nerve stimulation (TENS) has been studied and used for treating various types of pain and related symptoms [10]. In previous studies, TENS was shown to relieve pain effectively in cases of musculoskeletal pain, arthritic pain, low back pain, neuropathic pain, and postoperative pain [11–15]. Further, some studies have reported that the application of TENS improves motor function [15, 16]. In “the gate control theory,” which is the basic mechanism underlying the effect of TENS, electrical currents close the “gate” in the substantia gelatinosa of the dorsal horn by stimulating large-diameter fibers (A-beta) that inhibit small-diameter fibers (A-delta and C) [17]. Low-frequency (<10 Hz), high-intensity TENS induces analgesia by inhibiting pain transmission through the recruitment of descending inhibition mechanisms and is more frequently used for the treatment of chronic pain, while high-frequency TENS (80–100 Hz) is more often used for acute pain since it activates the gate control by stimulating A-beta fibers [18]. However, various inconsistencies exist among previous studies with respect to the therapeutic effects according to the frequency used [19]. To the best of our knowledge, no study has investigated the effect continuance time of high-frequency TENS for WMSDs. In addition, because of the varieties of work environments of the subjects included in previous studies, it remains unclear whether TENS treatment can benefit dental professionals with WMSDs.

Therefore, we investigated the immediate and short-term effects of a single session high-frequency TENS on work-related pain, fatigue, and range of motion in dental professionals. This study aimed to improve the quality of life and job skills of professionals in the field of dental health.

## 2. Material and Methods

### 2.1. Participants

This study was designed as a single-blind randomized placebo-controlled trial with six measurement time points: before work (pre-labor), after work (post-labor), measured immediately after TENS (post-TENS), and at 1 h, 3 h, and 1 day after TENS application. Overall, 47 dental professionals with WMSDs (13 dentists and 34 dental hygienists) participated in this study, which was conducted at 5 dental care centers at Incheon and Seoul in South Korea. They underwent an interview to record their medical history and physical examination by a blinded examiner. All participants met the following inclusion criteria: (1) 20–50 years of age, (2) employed for at least two years as a full-time worker, and (3) the pain-experienced subjects of neck and shoulder by labor for more than 2 months of subacute state. Patients with any history of cervical spinal or upper limb surgery, structural abnormality, severe musculoskeletal disability, or use of pacemaker were excluded. Further, subjects had not received or engaged in any other treatment before the study intervention. All participants were informed about the content of the project and its experimental purpose; written informed consent was obtained from all of them before participation in this study. All experimental conditions were approved by the Institutional Review Board of Gachon University. Sample size was calculated using G-Power version 3.1.3 (Informer Technologies, Dusseldorf, Germany). The effect size was set at 0.90; *α*-error at a probability of 0.05; and power at 0.85. A minimum of 21 participants were required in each group.

### 2.2. Experimental Procedure and Intervention

**Figure 1:**
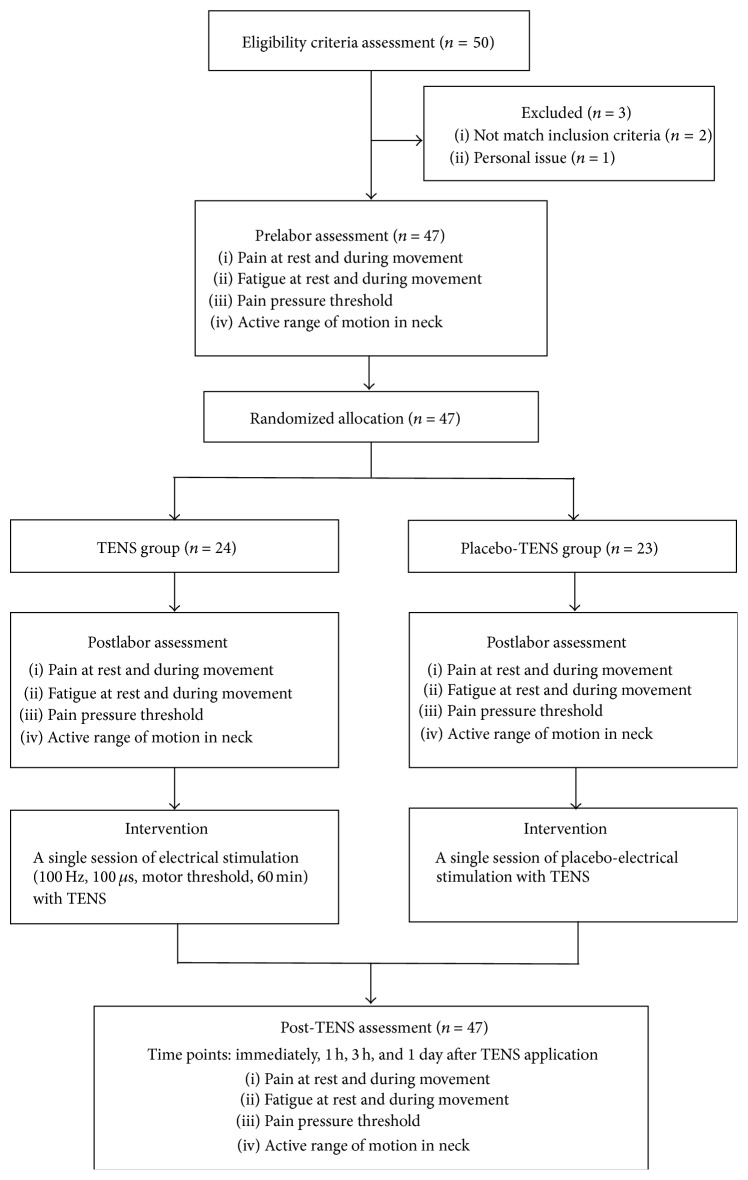
Experimental flow-diagram in this study.

A total of 50 participants volunteered for this study at initial recruitment. Three participants were not included for the following reasons: two participants did not satisfy the selection criteria and one failed to comply with the intervention for personal reasons. All experiment processes are shown in Figure 1. The participants were randomly assigned to the TENS group (*n* = 24) as the experimental group or the placebo-TENS group (*n* = 23) as the control group using a random allocation software by an independent examiner who was not involved in participant recruitment.

High-frequency TENS intervention (frequency 100 Hz, pulse width 100 *μ*s, motor threshold) was applied to tender trigger points of both the levator scapulae and trapezius muscles by a trained physical therapist to assure reliability. The intervention comprised a single session lasting for 60 min using a 2-channel TENS unit (TENS-7000; Koalaty Products Inc., USA). Applied stimulation usually evoked the occurrence of visual muscle contraction. While electrodes were attached at the same location, no electrical stimuli were administered in the placebo-TENS group. The investigator responded to any questions about the lack of TENS sensation in the placebo group by explaining that some types of TENS treatments are “barely perceptible.”

### 2.3. Outcome Measurements

General characteristics such as age, height, or weight were assessed using a survey method. The participants' pain and fatigue at rest and during movement, fatigue, pain pressure threshold (PPT), and active range of motion (AROM) were measured by a single, blinded examiner in order to reduce measurement error. All data were measured in the same place at six measurement time points. All outcome measurements at prelabor, postlabor, post-TENS, and 1 h, 3 h, and 1 day after TENS application were evaluated by the same examiner to ensure masking.

To assess pain intensity at rest and during movement, we used the 100 mm visual analog scale (VAS) with anchor words “no pain” at one end and “worst pain” at the other. The participants were instructed to mark their subjective pain intensity through the line at the appropriate represented point on the scale. Resting pain, defined by the VAS score at rest, was an unpleasant feeling or pain when patients were still. The VAS was also used to measure fatigue at rest and with movement. In fatigue measurement, the VAS consisted of a scale with the left end describing “no fatigue” and the right end described “worst fatigue imaginable.” The anchor words were “very sure it will not work” and “very sure it will work.” Movement-induced pain and fatigue, defined by the VAS score during movement, was an unpleasant feeling or pain and stiff sensation incurred by full-sagittal flexion of the head without compensatory movement of trunk [15, 20].

PPT was used to assess deep tissue hyperalgesia of the neck and upper extremity by using an algometer (Somedic AB, Farsta, Sweden) with a 1-cm probe [20, 21]. The purpose of the measurement was fully explained to the participants. The algometer was placed perpendicular to the planned sites. The examiner then applied pressure on the site at a consistent rate of 10 kPa/s. The participant was asked to inform the examiner when an unpleasant feeling or pain started. PPT was measured at sites in the levator scapulae and upper trapezius for assessing the effects of TENS at the painful site of stimulation.

AROM was evaluated using the distal inclinometer (Angle/Level, Dejon Tool Co., Covington, OH) for horizontal head rotation in the range without pain and fatigue sensation. The inclinometer's sensor was set at zero in maximal left rotation, followed by maximal right rotation when the subjects were in the neutral supine position. The angle was recorded when AROM of neck rotation reached the highest possible point without any compensatory lateral flexion with the head. This measurement was performed 3 times, and the mean value was recorded. Research has shown cervical AROM to be useful in the determination of function and for monitoring patient progress; further, it is reliable with good construct validity [22].

### 2.4. Data Analysis

Statistical analysis was performed using SPSS version 15.0 (SPSS Inc., Chicago, USA). The normal distributions of the results were tested by the Kolmogorov-Smirnov or Shapiro-Wilk test. Repeated-measures ANOVA was utilized to assess differences in VAS score, PPT, and AROM at the six measurement time points. Tukey's multiple comparison test was used as a post hoc test. The differences between the two groups were compared using an independent samples *t*-test or Mann-Whitney *U* test. Results were accepted as statistically significant at *P* < 0.05. The sample size was determined on the basis of the ability to detect a clinically significant improvement in the primary outcome measures of VAS score, PPT, and AROM.

## 3. Results

The demographic characteristics of all subjects are shown in Table 1. There were no significant differences between the TENS group and the placebo-TENS group (*P* > 0.05). Overall, 24 dental professionals with WMSDs received a single session high-frequency TENS and 23 received placebo-TENS in painful areas of the neck and shoulder. There were no dropouts in the study during the intervention period, and no statistical difference was noted at baseline between the groups (VAS score at rest and during movement, fatigue at rest and during movement, PPT at the levator scapulae and upper trapezius, and AROM; all *P* > 0.05). However, both groups showed significantly increased pain, fatigue, PPT, and AROM after work (postlabor; all *P* < 0.05).

The main findings of this study were as follows: (a) the TENS group showed a significantly greater reduction in pain at post-TENS and 1 h and 3 h after TENS application than the placebo-TENS group (Table 2). The VAS score at rest of the TENS group was reduced by 45% at post-TENS, 32% at 1 h after TENS application, and 49% at 3 h after TENS application, while VAS score during movement was decreased by 42% at 3 h after TENS application. (b) A significant decrease in the fatigue level at rest and during movement in the TENS group was observed at post-TENS and 1 h and 3 h after TENS application (Table 3); the greatest decrease in fatigue was observed at 3 h after TENS. (c) The outcomes of PPT at post-TENS and 1 h and 3 h after TENS application showed a significant decrease only in the TENS group (Table 4). In the levator scapulae, PPTs significantly improved by 24% at post-TENS, 24% at 1 h after TENS application, and 32% at 3 h after TENS application compared to pre-TENS treatment PPTs. In the upper trapezius, PPT significantly increased by 29% only at 3 h after TENS application. (d) AROM of head rotation in the TENS group was significantly more increased than that in the placebo group at post-TENS and at 1 h and 3 h after TENS application as short term effects (Table 5). In the TENS group, AROM immediately increased by 17% at post-TENS and by 15% at 1 h after TENS application. (e) The efficacy of TENS was maintained for 3 h after its application, but it did not persist until the following day (Tables 2–5), and (f) there was no placebo effect observed in the study (Tables 2–5).

## 4. Discussion

In dental professionals suffering from WMSDs, we found that the application of a single session high-frequency TENS immediately improved pain and fatigue at rest and during movement, PPT in the levator scapulae and upper trapezius, and AROM of horizontal head rotation. Additionally, these effects were maintained for 3 h after the intervention. The reason for no TENS effect in 1 day more is recovered on pain, fatigue, PPT, and AROM of both TENS and placebo-TENS groups within a day after finished labor. These results suggest that high-frequency TENS may have positive effects for relieving work-related pain and fatigue in dental professionals immediately.

According to previous studies related to the prevalence of WMSDs in dental professionals, over 60% of them reported at least one incident of musculoskeletal pain; this was considered to be caused by the physical load that placed them at risk for the occurrence of musculoskeletal disorders [5, 6]. The types of symptoms reported were pain, stiffness, or fatigue, and the regions of symptoms comprised the neck, wrist/hand, lower back, and shoulder [23]. These results are in tune with our present findings. For this study, when we conducted an initial survey of 70 dental professionals using a self-administered questionnaire for screening WMSDs, we found a high prevalence rate (71%), and 50 subjects had at least one work-related musculoskeletal symptom for the neck, shoulder, or hand/wrist. B. Valachi and K. Valachi who reviewed the implications of the specific nature of dental work on dentists' health and the potential for the development of musculoskeletal disorders reported that prolonged, seated working posture and repeated twisting of the spine, combined with excessive tightening of some tissues, could be the cause of various painful disorders and diseases of the musculoskeletal system in these professionals [24].

TENS is commonly used for the clinical research and activates a complex neuronal network, which results in a reduction in pain [25]. Possible mechanisms engaged in the effect of TENS on pain include the gate control theory, which specifies that the stimulation of nonnociceptive large-diameter afferents (A*β* fibers) inhibits nociceptive signal transmission and increases endogenous opioid release [17, 26]. High-frequency TENS has been proposed to modulate pain; however, the mechanisms underlying the resulting analgesia remain poorly understood. According to an animal study, high-frequency TENS decreases arthritis pain through delta opioid receptors in the spinal cord, while low-frequency TENS relieves pain through *μ*-opioid receptors; further, high-frequency TENS decreases pain by enhancing the release of the inhibitory neurotransmitter gamma-aminobutyric acid (GABA) in the dorsal horn of the spinal cord [27, 28]. A recent human study investigated the possible contribution of opioid receptors to analgesia induced by high-frequency TENS in acute skin pain [29]. Based on these reports, we hypothesize that high-frequency TENS affects and improves pain in WMSDs by increasing opioid and GABA release as well as by stimulating opioid receptors. Also, a randomized study that examined short-term pain relief with TENS found that the duration of pain relief following cessation of TENS was from 30 min to 2 h when stimulated time applied within 30 min [30, 31]. We assumed that the period for which the TENS effects are sustained depends on the duration of TENS application because high-frequency electrical stimulation is known to influence change activation in different regions of the brain according to the duration of application [32].

We applied high-frequency TENS on the tender trigger points of the neck and shoulder muscles, and our results showed significant improvements in pain and fatigue in the TENS group. These results are in tune with those of previous studies that examined this therapy. Carbonario et al. applied high-frequency TENS on bilateral tender points in the trapezium and supraspinatus in fibromyalgia patients who experienced improvements in pain, work performance, fatigue, stiffness, anxiety, and depression [18]. Further, high-frequency TENS applied to capsaicin-induced pain in dermatomes showed significantly reduced sensation to noxious stimulation following 60-min application [33]. However, these studies did not compare results against a placebo group and included a comparatively small number of study subjects. In clinical research, it is crucial to clarify the presence of a placebo effect. No electrical-stimulated pad of TENS may influence the pain response with activation of mechanoreceptors by light pressured attachment. In a randomized placebo-controlled trial, Gemmell and Hilland investigated the immediate effect of TENS in treating latent upper trapezius trigger points by measuring PPT; a statistically significant reduction in pain was observed in the TENS group, while no improvement was noted in the placebo group [34]. These results are similar to our study because most subjects in the TENS group showed a significant improvement in the PPT as well as no effect on placebo group. Therefore, we consider that high-frequency TENS may have inhibitory effects on work-induced pain in dental professionals.

AROM measurement indicates pure muscle ability in a movement range without pain and fatigue. In normal state, AROM angle of horizontal neck rotation previously reported 145° to 160°, while value of people with neck pain is below 130°. Our experiments of WMSDs in dental professionals also well showed pain-generated situation on time of before and after labor. Importantly, pain arising from muscle tenderness can decrease functional muscle contraction [35]. In our result, TENS application in the levator scapulae and upper trapezius decreased muscle tenderness based on the PPT results. AROM correlated significantly with the VAS score with movement (test: *r* = 0.828, *P* < 0.001) and PPT at the levator scapulae (test: *r* = 0.702, *P* < 0.001) and PPT at the upper trapezius (test: *r* = 0.657, *P* < 0.001). This result was similar to that of TENS improving AROM and pain in subjects suffering from shoulder pain [36]. Thus, TENS might contribute to improvements in functional muscle capability by reducing pain.

Pain is evaluated by the VAS, since it is the easiest approach tool to implement for subjects in the resting position [37]. In subjects in whom multiple joints and areas are affected, movement can increase pain due to connections between the bone and ligament as well as other connective tissues. In case of subjects with nonsevere musculoskeletal disorders, VAS score at rest can reflect lower levels of pain than that during movement. In our study, pain during movement had higher VAS scores than those in the resting position; these results were also reflected in the VAS results for fatigue. Furthermore, pain and fatigue during movement improved with TENS application. These findings indicated that high-frequency TENS was effective for both pain or fatigue-induced specific region and adjacent connective tissues.

Professionals such as dental hygienists and dentists experience fatigue because of progressively increasing tension to the neck and shoulder muscles during functional activities due to repetitive movements [38]. In a previous study, TENS increased the local blood flow in muscles, improved tissue oxygenation, and suppressed sympathetic tone in small arterioles [39, 40]; these changes aided muscle relaxation. Further, TENS application has been shown to cause relaxation of the upper trapezius in computer workers using electromyography as well as to decrease fatigue during movement in patients of fibromyalgia [34, 41]. Therefore, we consider that TENS might decrease fatigue via muscle relaxation in the upper extremity.

This study demonstrated that high-frequency TENS had positive effects on reducing pain and fatigue at rest and movement and increasing the PPT and AROM in dental professionals and provided evidence that the application of TENS would help dental professionals to restore and improve their job performance and quality of life. This study involved a single trial and measurements at six time points; however, the follow-up period was too short to examine the long-term effects of the therapy. Also, we did not use various parameters. Accordingly, further studies with high methodological quality, a variety of TENS modules, and a long-term study period are needed to assess the effects of TENS in the treatment of professionals with WMSDs.

## 5. Conclusion

Based on the results of this study, a single session high-frequency TENS may be effective in improving WMSDs in dental professionals. High-frequency TENS may play a positive role in reducing musculoskeletal pain and fatigue as well as increasing functional mobility in workers with WMSDs immediately. Our results support the notion of using TENS in the treatment of work-related disease or injury.

## Figures and Tables

**Table 1:** General characteristics of the participants.

	TENS group (*n* = 24)	Placebo-TENS group (*n* = 23)	*P*
Gender (male/female)	5/19	6/17	0.679
Job (dentist/dental hygienist)	6/18	7/16	0.707
Age (years)	30.7 ± 8.32	29.7 ± 8.47	0.685
Height (cm)	163.4 ± 8.16	165.4 ± 7.15	0.393
Weight (kg)	57.8 ± 9.13	58.9 ± 13.33	0.737

Values are expressed as mean ± standard deviation (SD).

**Table 2:** The changes in the visual analogue scale scores (VAS) in pain at rest and during full-sagittal neck movement after application of a single session TENS in postlabor state.

	TENS group (*n* = 24)	Placebo-TENS group (*n* = 23)	*P*
VAS score at rest (mm)			
Prelabor	7.0 ± 7.33^#^	6.4 ± 7.42^#^	0.794
Postlabor	34.2 ± 13.41^*∗*^	32.4 ± 18.46^*∗*^	
Post-TENS	18.7 ± 7.46^*∗*,#^	30.7 ± 17.67^*∗*^	
1 H TENS	23.2 ± 7.80^*∗*,#^	29.1 ± 16.00^*∗*^	
3 H TENS	17.3 ± 8.34^*∗*,#^	22.6 ± 12.87^*∗*^	
1 D TENS	7.9 ± 6.74^#^	8.2 ± 7.16^#^	
*P*	0.000	0.000	
Postlabor–prelabor	27.2 ± 11.26	26.0 ± 15.83	0.763
Post-TENS–postlabor	15.5 ± 9.10	1.7 ± 3.88	0.000
1 H TENS–postlabor	11.0 ± 6.96	3.3 ± 3.88	0.000
3 H TENS–postlabor	16.9 ± 7.78	9.8 ± 6.82	0.002
1 D TENS–postlabor	26.3 ± 10.86	24.2 ± 14.99	0.597

VAS score during movement (mm)			
Prelabor	17.7 ± 10.83^#^	17.4 ± 12.69^#^	0.927
Postlabor	48.8 ± 12.00^*∗*^	47.8 ± 19.06^*∗*^	
Post-TENS	33.5 ± 8.27^*∗*,#^	45.7 ± 17.60^*∗*^	
1 H TENS	37.3 ± 9.09^*∗*,#^	42.0 ± 15.86^*∗*^	
3 H TENS	28.4 ± 8.51^*∗*,#^	36.1 ± 14.92^*∗*^	
1 D TENS	18.1 ± 9.76^#^	18.7 ± 12.90^#^	
*P*	0.000	0.000	
Postlabor–prelabor	31.3 ± 12.45	30.4 ± 18.34	0.864
Post-TENS–postlabor	15.4 ± 8.25	2.2 ± 3.94	0.000
1 H TENS–postlabor	12.0 ± 6.70	5.9 ± 4.92	0.002
3 H TENS–postlabor	19.8 ± 7.76	11.7 ± 7.17	0.002
1 D TENS–postlabor	31.1 ± 11.28	29.1 ± 17.43	0.655

Values are expressed as mean ± standard deviation (SD).

*∗* indicates a significant difference within a group as compared to the prelabor value (*P* < 0.05).

# indicates a significant difference as compared to the postlabor value (*P* < 0.05).

VAS: visual analogue scale; 1 H TENS: 1 h after the application of transcutaneous electrical nerve stimulation (TENS); 3 H TENS: 3 h after TENS application; 1 D TENS: 1 day after TENS application.

**Table 3:** The changes in the visual analogue scale scores (VAS) in stiff sensation of muscles (fatigue) at rest and during full-sagittal neck movement after application of a single session TENS in postlabor state.

	TENS group (*n* = 24)	Placebo-TENS group (*n* = 23)	*P*
VAS score at rest (mm)			
Prelabor	11.0 ± 10.63^#^	12.4 ± 11.07^#^	0.672
Postlabor	41.0 ± 11.79^*∗*^	42.8 ± 19.06^*∗*^	
Post-TENS	24.3 ± 9.96^*∗*,#^	40.0 ± 16.65^*∗*^	
1 H TENS	28.3 ± 8.17^*∗*,#^	37.0 ± 15.58^*∗*^	
3 H TENS	21.5 ± 8.88^*∗*,#^	29.8 ± 12.75^*∗*,#^	
1 D TENS	12.1 ± 8.20^#^	14.4 ± 8.70^#^	
*P*	0.000	0.000	
Postlabor–prelabor	30.0 ± 13.27	30.4 ± 16.92	0.922
Post-TENS–postlabor	16.8 ± 7.86	2.8 ± 4.96	0.000
1 H TENS–postlabor	12.7 ± 6.08	5.9 ± 5.57	0.000
3 H TENS–postlabor	19.6 ± 8.07	13.0 ± 8.49	0.010
1 D TENS–postlabor	29.0 ± 10.32	28.5 ± 15.41	0.900

VAS score during movement (mm)			
Prelabor	24.4 ± 12.96^#^	25.4 ± 14.69^#^	0.794
Postlabor	60.5 ± 14.22^*∗*^	58.7 ± 17.85^*∗*^	
Post-TENS	41.2 ± 12.18^*∗*,#^	56.1 ± 16.16^*∗*^	
1 H TENS	42.3 ± 11.79^*∗*,#^	52.0 ± 15.58^*∗*^	
3 H TENS	34.2 ± 10.70^#^	45.4 ± 15.22^*∗*^	
1 D TENS	22.5 ± 12.77^#^	25.9 ± 13.71^#^	
*P*	0.000	0.000	
Postlabor–prelabor	36.1 ± 11.15	33.3 ± 14.12	0.445
Post-TENS–postlabor	19.3 ± 7.85	2.6 ± 3.33	0.000
1 H TENS–postlabor	18.2 ± 6.16	6.74 ± 4.16	0.000
3 H TENS–postlabor	26.3 ± 7.14	13.3 ± 4.16	0.000
1 D TENS–postlabor	38.0 ± 9.53	32.8 ± 11.76	0.104

Values are expressed as mean ± standard deviation (SD).

*∗* indicates a significant difference within a group as compared to the prelabor value (*P* < 0.05).

# indicates a significant difference as compared to the post-labor value (*P* < 0.05).

VAS: visual analogue scale; 1 H TENS: 1 h after the application of transcutaneous electrical nerve stimulation (TENS); 3 H TENS: 3 h after TENS application; 1 D TENS: 1 day after TENS application.

**Table 4:** The differences between pressure pain threshold (PPT) between after application of single TENS and no electrical stimulation in both the levator scapulae and the upper trapezius after labor.

	TENS group (*n* = 24)	Placebo-TENS group (*n* = 23)	*P*
Levator scapulae (N)			
Prelabor	51.1 ± 13.27^#^	51.6 ± 10.02^#^	0.739
Postlabor	35.9 ± 13.46^*∗*^	37.2 ± 13.13^*∗*^	
Post-TENS	44.5 ± 12.48^*∗*,#^	38.7 ± 13.81^*∗*^	
1 H TENS	44.4 ± 13.80^*∗*,#^	40.4 ± 13.31^*∗*^	
3 H TENS	47.0 ± 13.08^*∗*,#^	44.7 ± 12.56	
1 D TENS	52.3 ± 14.71^#^	51.2 ± 10.46^#^	
*P*	0.000	0.000	
Postlabor–prelabor	15.2 ± 8.42	14.4 ± 9.32	0.764
Post-TENS–postlabor	8.5 ± 5.41	1.5 ± 2.61	0.000
1 H TENS–postlabor	8.5 ± 5.37	3.2 ± 3.01	0.000
3 H TENS–postlabor	11.1 ± 5.93	7.5 ± 4.02	0.018
1 D TENS–postlabor	16.3 ± 6.92	14.0 ± 8.32	0.301

Upper trapezius (N)			
Prelabor	44.8 ± 12.48^#^	46.8 ± 10.40^#^	0.558
Postlabor	32.9 ± 13.07^*∗*^	33.6 ± 11.10^*∗*^	
Post-TENS	39.8 ± 12.31^*∗*^	34.4 ± 10.83^*∗*^	
1 H TENS	38.4 ± 12.51^*∗*^	35.5 ± 10.84^*∗*^	
3 H TENS	42.4 ± 12.37^#^	38.4 ± 10.15^*∗*^	
1 D TENS	46.4 ± 13.50^#^	46.3 ± 9.65^#^	
*P*	0.000	0.000	
Postlabor–prelabor	11.9 ± 8.15	13.2 ± 8.19	0.591
Post-TENS–postlabor	6.9 ± 5.73	0.8 ± 1.80	0.000
1 H TENS–postlabor	5.5 ± 4.69	1.9 ± 2.68	0.002
3 H TENS–postlabor	9.5 ± 6.52	4.8 ± 3.53	0.004
1 D TENS–postlabor	13.5 ± 6.77	12.7 ± 6.86	0.682

Values are expressed as mean ± standard deviation (SD).

*∗* indicates a significant difference within a group as compared to the prelabor value (*P* < 0.05).

# indicates a significant difference as compared to the postlabor value (*P* < 0.05).

1 H TENS: 1 h after the application of transcutaneous electrical nerve stimulation (TENS); 3 H TENS: 3 h after TENS application; 1 D TENS: 1 day after TENS application.

**Table 5:** The changes in active range of motion of horizontal head rotation after application of single TENS in postlabor state without pain or fatigue sense.

	TENS group (*n* = 24)	Placebo-TENS group (*n* = 23)	*P*
Horizontal head rotation (angle)			
Prelabor	156.3 ± 13.29^#^	151.3 ± 13.59^#^	0.989
Postlabor	119.8 ± 26.64^*∗*^	121.3 ± 29.32^*∗*^	
Post-TENS	139.6 ± 15.10^*∗*,#^	125.0 ± 24.40^*∗*^	
1 H TENS	137.3 ± 17.51^*∗*,#^	128.3 ± 25.12^*∗*^	
3 H TENS	145.2 ± 14.41^#^	137.0 ± 20.82^*∗*^	
1 D TENS	157.5 ± 10.84^#^	155.4 ± 12.78^#^	
*P*	0.000	0.000	
Postlabor–prelabor	36.5 ± 23.84	35.0 ± 25.85	0.841
Post-TENS–postlabor	19.8 ± 13.95	3.7 ± 6.78	0.000
1 H TENS–postlabor	17.5 ± 12.51	7.0 ± 7.65	0.001
3 H TENS–postlabor	25.4 ± 16.35	15.7 ± 11.11	0.021
1 D TENS–postlabor	37.7 ± 22.84	34.3 ± 24.39	0.606

Values are expressed as mean ± standard deviation (SD).

*∗* indicates a significant difference within a group as compared to the prelabor value (*P* < 0.05).

# indicates a significant difference as compared to the postlabor value (*P* < 0.05).

1 H TENS: 1 h after the application of transcutaneous electrical nerve stimulation (TENS); 3 H TENS: 3 h after TENS application; 1 D TENS: 1 day after TENS application.
